# Walnut Consumption Is Associated with Lower Risk of Type 2 Diabetes in Women^1^,^2^

**DOI:** 10.3945/jn.112.172171

**Published:** 2013-02-20

**Authors:** An Pan, Qi Sun, JoAnn E. Manson, Walter C. Willett, Frank B. Hu

**Affiliations:** 3 Department of Nutrition, and; 4 Department of Epidemiology, Harvard School of Public Health, Boston, MA; 5 Saw Swee Hock School of Public Health and Yong Loo Lin School of Medicine, National University of Singapore and National University Health System, Singapore; and; 6 Channing Division of Network Medicine, and; 7 Division of Preventive Medicine, Department of Medicine, Brigham and Women’s Hospital and Harvard Medical School, Boston, MA

## Abstract

Walnuts are rich in polyunsaturated fatty acids and have been shown to improve various cardiometabolic risk factors. We aimed to investigate the association between walnut intake and incident type 2 diabetes in 2 large cohort studies: the Nurses’ Health Study (NHS) and NHS II. We prospectively followed 58,063 women aged 52–77 y in NHS (1998–2008) and 79,893 women aged 35–52 y in NHS II (1999–2009) without diabetes, cardiovascular disease, or cancer at baseline. Consumption of walnuts and other nuts was assessed every 4 y using validated food frequency questionnaires. Self-reported type 2 diabetes was confirmed by a validated supplemental questionnaire. We documented a total of 5930 incident type 2 diabetes cases during 10 y of follow-up. In the multivariable-adjusted Cox proportional hazards model without body mass index (BMI), walnut consumption was associated with a lower risk of type 2 diabetes, and the HRs (95% CIs) for participants consuming 1–3 servings/mo (1 serving = 28 g), 1 serving/wk, and ≥2 servings/wk of walnuts were 0.93 (0.88–0.99), 0.81 (0.70–0.94), and 0.67 (0.54–0.82) compared with women who never/rarely consumed walnuts (*P*-trend < 0.001). Further adjustment for updated BMI slightly attenuated the association and the HRs (95% CIs) were 0.96 (0.90–1.02), 0.87 (0.75–1.01), and 0.76 (0.62–0.94), respectively (*P*-trend = 0.002). The consumption of total nuts (*P*-trend < 0.001) and other tree nuts (*P*-trend = 0.03) was also inversely associated with risk of type 2 diabetes, and the associations were largely explained by BMI. Our results suggest that higher walnut consumption is associated with a significantly lower risk of type 2 diabetes in women.

## Introduction

Diabetes is estimated to affect 25.6 million American adults (1) and 366 million people worldwide (2), and the numbers will continue to increase to ∼552 million by 2030 globally (2). Type 2 diabetes makes up >90% of all diabetes cases. Therefore, primary prevention of type 2 diabetes through diet and lifestyle modifications is of paramount public health importance. Recent evidence suggests that the type of fat rather than total fat intake plays an important role in the development of type 2 diabetes (3, 4). Studies have shown that a higher intake of MUFAs and PUFAs and a lower intake of saturated fat and *trans* fat is associated with a reduced risk of type 2 diabetes (4).

The relationship between regular nut consumption and type 2 diabetes risk has attracted a great deal of attention. Although nuts are high in fats, most of the fats are MUFAs and PUFAs (5). Nuts also contain other bioactive compounds that appear to exert favorable effects on type 2 diabetes, including vegetable proteins, plant sterols, dietary fiber, and antioxidants (5). A previous analysis from the Nurses’ Health Study (NHS) reported an inverse relation between frequent nut consumption and risk of incident type 2 diabetes (6); however, the association with specific types of tree nuts has not been reported.

Despite many commonalities in nutrient contents, substantial variations in fatty acids content exist among nuts. For example, compared with other tree nuts, walnuts are uniquely high in PUFAs (47% in weight) (5), which comprise both n6 PUFAs (38%) and n3 PUFAs (α-linolenic acid, 18:3n3, 9%). Because of potential benefits of PUFAs in preventing diabetes, we specifically investigated the association between walnut consumption and risk of type 2 diabetes by using data from 2 prospective cohort studies, NHS and NHS II, with 10 y of follow-up.

## Methods

### 

#### Study population.

Details of the 2 cohorts were previously described (6, 7). The NHS consisted of 121,700 registered female nurses from 11 U.S. states who were enrolled in 1976. The NHS II was established in 1989 and was comprised of 116,671 younger female registered nurses from 14 states. The cohorts have been updated with biennial validated questionnaires to collect information on medical history, lifestyle, and health factors. The cumulative follow-up of both cohorts exceeds 90% of potential person-times. Women who answered the 1998 questionnaire in the NHS (*n* = 84,733, age range 52–77 y) and 1999 questionnaire in the NHS II (*n* = 87,499, age range 35–52 y) served as the baseline population for our current analyses, because the information on walnut consumption was first available in these years. Participants were excluded if they had diabetes, heart diseases, stroke, or cancer at baseline (*n* = 25,052 in the NHS and *n* = 6681 in the NHS II), implausible energy intake (<500 or >3500 kcal/d; *n* = 1179 in the NHS and *n* = 420 in the NHS II), missing information on nut variables (*n* = 373 in the NHS and *n* = 415 in the NHS II), or missing information on body weight (*n* = 66 in the NHS and *n* = 90 in the NHS II). These exclusions left a total of 58,063 women in the NHS and 79,893 women in the NHS II for this analysis. This study was approved by the institutional review boards of Brigham and Women’s Hospital and Harvard School of Public Health.

#### Assessment of consumption of walnuts and other nuts.

Dietary information was collected using validated FFQs with over 130 items that were administered to the participants every 4 y. All FFQs inquired about how often, on average, participants consumed each food using standard portion sizes over the past year. There were 9 possible responses, ranging from “never or less than once per month” to “six or more times per day.” Starting from 1986 in the NHS and 1991 in the NHS II, participants were asked about their usual intakes of peanuts and tree nuts using standard portion sizes (small packet or 1 oz, 28 g). The food item of walnuts was first introduced on the questionnaires in 1998 in the NHS and 1999 in the NHS II, along with peanuts and other tree nuts. Although peanuts are botanically classified as legumes, the fatty acid and nutrient contents of peanuts are similar to other tree nuts; therefore, we also included peanuts in the current analysis. The total nut consumption was the sum of walnuts (if available), peanuts, and other tree nuts. A validation study indicated a correlation coefficient of 0.75 between the FFQ and four 1-wk diet records for total nuts in the NHS (8).

#### Ascertainment of incident type 2 diabetes.

In both cohorts, a supplemental questionnaire regarding symptoms, diagnostic tests, and hypoglycemic therapy was mailed to participants who reported a diagnosis of diabetes on the main questionnaire. A case of type 2 diabetes was considered confirmed if at least one of the following was reported on the supplemental questionnaire according to the 1997 American Diabetes Association criteria (9): *1*) one or more classic symptoms (excessive thirst, polyuria, weight loss, hunger) plus fasting plasma glucose concentrations of at least 7.0 mmol/L or random plasma glucose concentrations of at least 11.1 mmol/L; *2*) at least 2 elevated plasma glucose concentrations on different occasions (fasting concentrations of at least 7.0 mmol/L, random plasma glucose concentrations of at least 11.1 mmol/L, and/or concentrations of at least 11.1 mmol/L after ≥2 h shown by oral glucose tolerance testing) in the absence of symptoms; or *3*) treatment with hypoglycemic medication (insulin or oral hypoglycemic agent).

A previous validation study in the NHS suggested that the self-reported type 2 diabetes diagnosis through supplemental questionnaire confirmation was highly accurate: of 62 type 2 diabetes cases who were confirmed by the questionnaire, 61 (98%) were reconfirmed by medical records (10). Moreover, in another substudy to assess the prevalence of undiagnosed diabetes in the NHS, only 1 (0.5%) of 200 women who did not report a previous diagnosis of diabetes had an elevated fasting plasma glucose or plasma fructosamine concentration in the diabetic range (11). Only type 2 diabetes cases confirmed by the supplemental questionnaires were included in the analysis.

#### Assessment of covariates.

In the biennial follow-up questionnaires, we inquired and updated information on risk factors for chronic diseases, such as body weight, cigarette smoking, physical activity, menopausal status, and hormone use. Other dietary variables included in the statistical models were alcohol intake, whole grains, fruit, vegetables, fish, red meat, coffee and sugar-sweetened beverages, which have been updated every 4 y and related to diabetes risk in our previous investigations. Information about family history of diabetes and race was also collected.

#### Statistical analysis.

Person-years for each participant were calculated from the return date of the baseline questionnaire to the date of diagnosis of type 2 diabetes, death, or the end of the follow-up period (June 30, 2008 for NHS and June 30, 2009 for NHS II), whichever came first. Given that the 2 cohorts were similar in study design and follow-up years, they were combined into one database for the current analysis. Time-dependent Cox proportional hazards models were used to estimate the HRs of developing type 2 diabetes according to walnut consumption categories (never/rarely, <1 serving/wk, 1 serving/wk, ≥2 servings/wk). In the multivariable analysis, we simultaneously controlled for various potential confounding factors, including age (continuous), questionnaire-cycle (each 2-y interval), race (white, non-white), family history of diabetes (yes, no), smoking status [never, past, current (1–14, 15–24, ≥25 cigarettes/d)], alcohol intake (0, 0.1–4.9, 5.0–14.9, ≥15.0 g/d), physical activity (<3.0, 3.0–8.9, 9.0–17.9, 18.0–26.9, ≥27.0 metabolic equivalent task-h/wk), postmenopausal status, and menopausal hormone use [premenopausal, postmenopausal (never, past, or current hormone use)], use of multivitamin (yes, no), total energy, and other dietary variables (whole grains, fruits, vegetables, fish, red meat, coffee, and sugar-sweetened beverages, all in quintiles). In additional analyses, we further adjusted for BMI (<23.0, 23.0–24.9, 25.0–29.9, 30.0–34.9, ≥35.0 kg/m^2^) to examine the degree to which the association between walnut consumption and type 2 diabetes was mediated by BMI (7). The above covariates were updated every 2 or 4 y using the most recent data for each 2-y follow-up interval.

To better represent long-term diet and minimize within-person variation, we created cumulative averages of food intakes from baseline to the censoring events (12). We stopped updating the dietary variables when the participants reported a diagnosis of stroke, myocardial infarction, angina, or cancer, because these conditions might lead to changes in dietary intakes (12). Missing values during the follow-up were replaced by the carry-forward method.

We also conducted a further analysis to evaluate the association between total nut and peanut intake and risk of type 2 diabetes in the 2 cohorts. In this particular analysis, we used the 1986 and 1991 questionnaire years as baseline for the NHS and NHS II, respectively. A total of 59,259 women in the NHS and 91,799 women in the NHS II were included. The analysis strategy was the same as in the walnut analysis; however, the 2 cohorts were analyzed separately, because the follow-up years were different. The results across the 2 cohorts were then pooled by an inverse variance-weighted, fixed-effect meta-analysis.

Tests for linear trend were conducted by treating the median value for each category as a continuous variable. All *P* values were 2-sided and data were analyzed with SAS 9.2 (SAS Institute).

## Results

The baseline characteristics according to the frequency of walnut consumption in the 2 cohorts are shown in **Table 1**. Women with more frequent walnut consumption were older and tended to weigh less, exercise more, and smoke less than women with infrequent consumption. Women who ate more walnuts also consumed more fish, whole grains, fruit and vegetables, and total energy. Consumption of walnuts was positively correlated with intakes of peanuts (Spearman correlation coefficient = 0.30) and other tree nuts (Spearman correlation coefficient = 0.40).

**TABLE 1:** Baseline characteristics according to walnut consumption categories in the 2 prospective cohorts^1^

		Frequency of walnut consumption				
Characteristic	Total sample	Never/rarely	<1 serving/wk	1 serving/wk	≥2 servings/wk	*P*-trend
*n*	137,956	107,333	24,022	4716	1885	—
Age, *y*	52.2 ± 11.1	51.9 ± 11.2	52.8 ± 10.6	53.6 ± 10.7	55.0 ± 11.2	<0.001
BMI, *kg/m^2^*	26.3 ± 5.6	26.3 ± 5.6	26.2 ± 5.5	26.0 ± 5.5	25.4 ± 5.2	<0.001
Physical activity, *metabolic equivalent task-h/wk*	18.5 ± 22.8	18.0 ± 22.5	19.9 ± 23.0	22.2 ± 25.9	23.7 ± 28.1	<0.001
Family history of diabetes, *n (%)*	43,178 (31.3)	33,565 (31.3)	7546 (31.4)	1471 (31.2)	596 (31.6)	0.73
Menopausal status, *n (%)*						<0.001
Premenopausal	66,226 (48.0)	52,865 (49.3)	10,645 (44.3)	1998 (42.4)	718 (38.1)	
Postmenopausal, no hormone use	14,295 (10.4)	11,043 (10.3)	2510 (10.5)	514 (10.9)	228 (12.1)	
Postmenopausal, past hormone use	19,162 (13.9)	14,658 (13.7)	3468 (14.4)	703 (14.9)	333 (17.7	
Postmenopausal, current hormone use	29,442 (21.3)	21,798 (20.3)	5954 (24.8)	1204 (25.5)	486 (25.8)	
Missing value	8831 (6.4)	6969 (6.5)	1445 (6.0)	297 (6.3)	120 (6.4)	
History of hypertension, *n (%)*	33,470 (24.3)	26,189 (24.4)	5729 (23.9)	1104 (23.4)	448 (23.8)	<0.001
History of hypercholesterolemia, *n (%)*	52,174 (37.8)	40,347 (37.6)	9197 (38.3)	1843 (39.1)	787 (41.8)	48.1
Current smoker, *n (%)*	13,332 (9.7)	10,910 (10.2)	2005 (8.4)	317 (6.7)	100 (5.3)	<0.001
White, *n (%)*	133,828 (97.0)	104,104 (97.0)	23,339 (97.2)	4577 (97.1)	1808 (95.9)	0.56
Multivitamin use, *n (%)*	67,124 (48.7)	51,650 (48.1)	11,890 (49.5)	2532 (53.7)	1052 (55.8)	<0.001
Alcohol consumption, *g/d*	4.5 ± 8.2	4.5 ± 8.2	4.8 ± 8.0	4.9 ± 7.6	4.6 ± 8.2	0.10
Total energy intake, *kcal/d*	1790 ± 558	1730 ± 540	1980 ± 555	2100 ± 585	2160 ± 577	<0.001
Whole grain intake, *g/d*	27.5 ± 18.2	27.3 ± 18.4	27.7 ± 17.0	28.7 ± 17.2	30.1 ± 19.6	<0.001
Red/processed meat intake, *g/d*	60.6 ± 45.4	59.7 ± 44.6	64.6 ± 46.1	64.1 ± 55.1	56.2 ± 54.9	0.01
Fish intake, *g/d*	18.9 ± 19.0	17.8 ± 18.3	22.1 ± 19.7	25.2 ± 23.3	25.4 ± 26.9	<0.001
Poultry intake, *g/d*	58.5 ± 45.1	57.8 ± 44.8	60.3 ± 43.7	63.1 ± 55.5	59.1 ± 52.5	<0.001
Vegetable intake, *g/d*	245 ± 153	232 ± 145	281 ± 160	312 ± 192	341 ± 216	<0.001
Fruit intake, *g/d*	161 ± 124	152 ± 121	184 ± 125	209 ± 137	229 ± 166	<0.001
Coffee, *g/d*	389 ± 372	389 ± 372	398 ± 375	393 ± 375	375 ± 382	0.79
Sugar-sweetened beverage, *g/d*	158 ± 302	162 ± 310	151 ± 281	137 ± 263	122 ± 266	<0.001
Fiber, *g/d*	19.7 ± 6.0	19.4 ± 5.9	20.3 ± 5.8	21.6 ± 6.5	22.9 ± 7.2	<0.001
Glycemic load	122 ± 24	123 ± 24	118 ± 22	115 ± 23	107 ± 28	<0.001
Total fats, *g/d*	57.3 ± 14.2	56.6 ± 14.2	59.1 ± 13.4	60.7 ± 15.1	66.4 ± 18.6	<0.001
PUFA to SFA ratio	0.58 ± 0.20	0.58 ± 0.20	0.65 ± 0.21	0.72 ± 0.24	1.07 ± 0.46	<0.001
α-Linolenic acid, *g/d*	1.00 ± 0.36	0.93 ± 0.31	1.13 ± 0.29	1.24 ± 0.30	2.08 ± 0.73	<0.001
Magnesium, *mg/d*	348 ± 97	345 ± 98	355 ± 92	369 ± 96	399 ± 106	<0.001
Arginine, *g/d*	4.04 ± 0.85	4.03 ± 0.86	4.06 ± 0.79	4.14 ± 0.88	4.33 ± 1.04	<0.001
Walnut intake, *g/d*	0.56 ± 2.24	0 ± 0	1.96 ± 0	3.92 ± 0	16.2 ± 1.96	<0.001
Peanut intake, *g/d*	1.40 ± 3.92	1.12 ± 3.36	2.24 ± 0.15	3.92 ± 5.60	5.88 ± 9.24	<0.001
Other nut intake, *g/d*	1.12 ± 3.64	0.84 ± 3.36	1.96 ± 3.92	3.64 ± 5.60	7.84 ± 9.52	<0.001
Total nut intake, *g/d*	3.36 ± 3.92	1.96 ± 5.04	5.88 ± 6.16	3.92 ± 8.12	28.0 ± 13.4	<0.001

1 Data are mean ± SD or *n* (%) as specified. 1 serving of walnuts = 28 g.

We documented a total of 5930 incident type 2 diabetes cases (3166 in the NHS and 2764 cases in the NHS II) during 10 y of follow-up. As shown in **Table 2**, walnut consumption was inversely associated with risk of type 2 diabetes. In the multivariable-adjusted model without BMI, the pooled HRs (95% CIs) for participants consuming 1–3 servings/mo, 1 serving/wk, and ≥2 servings/wk of walnuts were 0.93 (0.88–0.99), 0.81 (0.70–0.94), and 0.67 (0.54–0.82), respectively, compared with women who never/rarely consumed walnuts (*P*-trend < 0.001). Further adjustment for updated BMI slightly attenuated the association, and the HRs (95% CIs) were 0.96 (0.90–1.02), 0.87 (0.75–1.01), and 0.76 (0.62–0.94), respectively (*P*-trend = 0.002). Each 2-servings/wk increment of walnut intake was associated with 21% (13–29%) and 15% (6–23%) lower risk of incident type 2 diabetes before and after adjustment for BMI, respectively. The associations were similar to adjustments for saturated fat, *trans* fat, glycemic load, and cereal fiber instead of adjustment for food variables, and further adjustment for PUFAs, α-linolenic acid, total fiber, magnesium, and arginine did not change the results (data not shown). We did not find any interaction between walnut consumption and obesity status, physical activity, dietary quality, and family history of diabetes in the risk of type 2 diabetes (data not shown). For other tree nuts, we also found an inverse association with risk of type 2 diabetes. In the multivariable-adjusted model without BMI, the pooled HRs (95% CIs) for participants consuming 1–3 servings/mo, 1 serving/wk, and ≥2 servings/wk of other tree nuts were 0.99 (0.94–1.06), 0.93 (0.83–1.04), and 0.88 (0.77–0.99) compared with women who never/rarely consumed other tree nuts (*P*-trend = 0.03). However, the association was attenuated to null after further adjustment for BMI.

**TABLE 2:** Relationships between walnut consumption and risk of type 2 diabetes in the 2 prospective cohorts of women^1^

	Frequency of walnut consumption					
	Never/rarely	<1 serving/wk	1 serving/wk	≥2 servings/wk	*P*-trend	HR (95% CI) for 2 servings/wk
Walnuts				
Cases/person-years	4224/91,6280	1433/320,434	183/49,687	90/29,180		5930/131,5581
Age-adjusted model	1.00	0.90 (0.84–0.95)	0.75 (0.64–0.87)	0.61 (0.49–0.75)	<0.001	0.73 (0.66–0.81)
Multivariable model*^2^*	1.00	0.93 (0.88–0.99)	0.81 (0.70–0.94)	0.67 (0.54–0.82)	<0.001	0.79 (0.71–0.87)
Multivariable model + BMI*^3^*	1.00	0.96 (0.90–1.02)	0.87 (0.75–1.01)	0.76 (0.62–0.94)	0.002	0.85 (0.77–0.94)
Other tree nuts				
Cases/person-years	3672/79,5074	1624/355,405	349/88,720	285/76,381		5930/131,5581
Age-adjusted model	1.00	0.96 (0.91–1.02)	0.84 (0.75–0.94)	0.78 (0.69–0.88)	<0.001	0.90 (0.85–0.95)
Multivariable model*^2^*	1.00	0.99 (0.94–1.06)	0.93 (0.83–1.04)	0.88 (0.77–0.99)	0.03	0.94 (0.90–0.99)
Multivariable model + BMI*^3^*	1.00	1.01 (0.95–1.08)	1.01 (0.90–1.13)	1.04 (0.92–1.18)	0.49	1.02 (0.97–1.07)

1 Data are based on a pooled database of 10 y of follow-up in the NHS (1998–2008) and NHS II (1999–2009). 1 serving of walnuts = 28 g. NHS, Nurses’ Health Study.

2 Multivariable model: adjusted for age (continuous), race (white, non-white), family history of diabetes (yes, no), smoking status [never, past, current (1–14, 15–24, ≥25 cigarettes/d)], alcohol intake (0, 0.1–4.9, 5.0–14.9, ≥15.0 g/d), physical activity (<3.0, 3.0–8.9, 9.0–17.9, 18.0–26.9, ≥27.0 metabolic equivalent task-h/wk), postmenopausal status and menopausal hormone use [premenopausal, postmenopausal (no, past, or current hormone use)], use of multivitamin (yes, no), total energy intake, and other dietary variables (all in quintiles), including whole grains, fruits, vegetables, fish, red meat, coffee, and sugar-sweetened beverages.

3 Multivariable model + BMI: <23.0, 23.0–24.9, 25.0–29.9, 30.0–34.9, ≥35 kg/m^2^.

We further examined the relation of total nut (including peanut, walnut, and other nuts) and peanut intakes with risk of type 2 diabetes (**Table 3**). Total nut consumption was associated with a lower risk of incident type 2 diabetes before adjustment for BMI in both cohorts. In the pooled analysis, the HRs (95% CIs) for participants consuming 1–3 servings/mo, 1 serving/wk, 2–4 servings/wk, and ≥5 servings/wk of total nuts were 0.96 (0.92–1.01), 0.95 (0.89–1.02), 0.89 (0.80–0.98), and 0.84 (0.75–0.93), respectively, compared with women who never/rarely consumed nuts (*P*-trend < 0.001). However, the association was attenuated to null after adjustment for BMI (*P*-trend = 0.95). Frequent consumption of total tree nuts was also associated with a trend toward a lower risk of incident type 2 diabetes before adjustment for BMI (HR = 0.85; 95% CI: 0.75–0.95; comparing ≥2 servings/wk vs. never/rarely; *P*-trend = 0.054), but not after adjustment for BMI. There was also an inverse trend for peanut consumption before adjustment for BMI, but the association became nonsignificant after further adjustment for BMI.

**TABLE 3:** Cohort-specific and pooled results for the relationships between total nuts and peanut consumption and risk of type 2 diabetes in the 2 prospective cohorts of women^1^

	Frequency of nut consumption						
NHS (1986–2008)	Never/rarely	<1 serving/wk	1 serving/wk	2–4 servings/wk	≥5 servings/wk	*P*-trend	HR (95% CI) for 2 servings/wk
Total nuts					
Cases/person-years	1713/370,858	2117/479,539	694/151,885	318/82,750	279/79,216		5121/116,4248
Age-adjusted model	1.00	0.92 (0.87–0.98)	0.90 (0.82–0.98)	0.80 (0.71–0.90)	0.74 (0.65–0.84)	<0.001	0.90 (0.86–0.94)
Multivariable model^2^	1.00	0.97 (0.91–1.04)	1.00 (0.91–1.09)	0.91 (0.80–1.02)	0.87 (0.77–0.99)	0.051	0.96 (0.92–1.00)
Multivariable model + BMI^3^	1.00	0.99 (0.92–1.05)	1.05 (0.96–1.15)	0.98 (0.87–1.11)	1.00 (0.87–1.14)	0.84	1.00 (0.96–1.05)
Total tree nuts				
Cases/person-years	3204/691,101	1368/334,154	331/80,486	218/58,506			5121/116,4248
Age-adjusted model	1.00	0.95 (0.89–1.02)	0.91 (0.81–1.02)	0.75 (0.66–0.87)		0.002	0.91 (0.86–0.97)
Multivariable model^2^	1.00	1.01 (0.95–1.08)	1.01 (0.90–1.14)	0.91 (0.79–1.05)		0.70	0.99 (0.94–1.05)
Multivariable model + BMI^3^	1.00	1.03 (0.96–1.10)	1.07 (0.95–1.19)	1.00 (0.86–1.15)		0.28	1.03 (0.98–1.09)
Peanuts				
Cases/person-years	2415/532,763	1953/438,313	471/115,823	282/77,349			5121/116,4248
Age-adjusted model	1.00	0.99 (0.93–1.05)	0.92 (0.83–1.01)	0.84 (0.75–0.95)		0.009	0.92 (0.87–0.98)
Multivariable model*^2^*	1.00	1.01 (0.95–1.07)	0.99 (0.89–1.09)	0.93 (0.82–1.06)		0.41	0.98 (0.92–1.03)
Multivariable model + BMI*^3^*	1.00	1.03 (0.97–1.09)	1.06 (0.95–1.17)	1.05 (0.92–1.19)		0.38	1.03 (0.97–1.09)
NHS II (1991–2009)						
Total nuts					
Cases/person-years	1691/714164	1676/622,298	414/147,139	185/66,544	132/49,522		4098/159,9667
Age-adjusted model	1.00	0.98 (0.91–1.05)	0.88 (0.79–0.98)	0.86 (0.74–1.00)	0.78 (0.65–0.93)	<0.001	0.88 (0.83–0.94)
Multivariable model^2^	1.00	0.95 (0.88–1.02)	0.89 (0.79–0.99)	0.86 (0.73–1.00)	0.77 (0.64–0.92)	<0.001	0.89 (0.83–0.94)
Multivariable model + BMI^3^	1.00	0.99 (0.92–1.06)	1.00 (0.89–1.12)	1.00 (0.86–1.17)	1.02 (0.85–1.23)	0.69	0.99 (0.93–1.05)
Total tree nuts				
Cases/person-years	2632/1,047,652	1185/438,580	200/77,904	81/35,532			4098/1,599,667
Age-adjusted model	1.00	0.96 (0.90–1.03)	0.82 (0.71–0.95)	0.68 (0.55–0.85)		<0.001	0.80 (0.71–0.89)
Multivariable model^2^	1.00	0.95 (0.89–1.02)	0.88 (0.76–1.02)	0.70 (0.56–0.91)		<0.001	0.82 (0.74–0.92)
Multivariable model + BMI^3^	1.00	1.00 (0.93–1.08)	1.03 (0.89–1.19)	0.94 (0.75–1.17)		0.68	0.98 (0.88–1.08)
Peanuts				
Cases/person-years	2303/940,712	1466/540,865	241/85,875	88/32,215			4098/1,599,667
Age-adjusted model	1.00	1.02 (0.96–1.09)	1.02 (0.89–1.17)	0.90 (0.73–1.12)		0.14	0.93 (0.83–1.03)
Multivariable model^2^	1.00	0.97 (0.90–1.03)	0.98 (0.86–1.12)	0.84 (0.68–1.04)		0.02	0.88 (0.79–0.98)
Multivariable model + BMI^3^	1.00	1.01 (0.94–1.08)	1.11 (0.97–1.27)	1.01 (0.81–1.25)		0.95	1.00 (0.90–1.12)
Pooled results^4^
Total nuts						
Age-adjusted model	1.00	0.95 (0.91–0.99)	0.89 (0.83–0.95)	0.82 (0.75–0.90)	0.75 (0.68–0.84)	<0.001	0.90 (0.87–0.93)
Multivariable model*^2^*	1.00	0.96 (0.92–1.01)	0.95 (0.89–1.02)	0.89 (0.80–0.98)	0.84 (0.75–0.93)	<0.001	0.93 (0.90–0.97)
Multivariable model + BMI*^3^*	1.00	0.99 (0.94–1.04)	1.03 (0.96–1.10)	0.99 (0.90–1.09)	1.01 (0.90–1.12)	0.95	1.00 (0.97–1.03)
Total tree nuts						
Age-adjusted model	1.00	0.96 (0.91–1.00)	0.87 (0.80–0.96)	0.73 (0.65–0.82)		<0.001	0.89 (0.84–0.93)
Multivariable model^2^	1.00	0.98 (0.94–1.03)	0.96 (0.88–1.05)	0.85 (0.75–0.95)		0.054	0.95 (0.91–1.00)
Multivariable model + BMI^3^	1.00	1.02 (0.97–1.07)	1.05 (0.96–1.15)	0.98 (0.87–1.10)		0.44	1.02 (0.97–1.07)
Peanuts						
Age-adjusted model	1.00	1.00 (0.96–1.05)	0.95 (0.88–1.03)	0.86 (0.77–0.96)		0.003	0.92 (0.88–0.97)
Multivariable model^2^	1.00	0.99 (0.95–1.04)	0.98 (0.91–1.07)	0.91 (0.81–1.01)		0.07	0.95 (0.90–1.00)
Multivariable model + BMI^3^	1.00	1.02 (0.97–1.06)	1.07 (0.99–1.17)	1.04 (0.93–1.16)		0.42	1.02 (0.97–1.07)

1 Data are based on 22 y of follow-up in the NHS (1986–2008), and 18 y of follow, up in the NHS II (1991–2009). 1 serving of walnuts = 28 g. NHS, Nurses’ Health Study.

2 Multivariable model: adjusted for age (continuous), race (white, non-white), family history of diabetes (yes, no), smoking status [never, past, current (1–14, 15–24, ≥25 cigarettes/d)], alcohol intake (0, 0.1–4.9, 5.0–14.9, ≥15.0 g/d), physical activity (<3.0, 3.0–8.9, 9.0–17.9, 18.0–26.9, ≥27.0 metabolic equivalent task-h/wk), postmenopausal status and menopausal hormone use [premenopausal, postmenopausal (no, past, or current hormone use)], use of multivitamin (yes, no), total energy intake, and other dietary variables (all in quintiles), including whole grains, fruits, vegetables, fish, red meat, coffee, and sugar-sweetened beverages.

3 Multivariable model + BMI: <23.0, 23.0–24.9, 25.0–29.9, 30.0–34.9, ≥35 kg/m^2^.

4 The results were pooled by a fixed-effect meta-analysis across the 2 cohorts.

## Discussion

In 2 large prospective cohorts of U.S. women, we found an inverse association between walnut consumption and risk of type 2 diabetes. This association was attenuated but remained significant after adjusting for BMI. Consistent with our previous analyses, regular consumption of peanut and tree nuts was also associated with a significantly lower risk of type 2 diabetes, but these associations were largely explained by body weight.

Compared with other nuts, which typically contain a high amount of monounsaturated fats, walnuts are unique because they are rich in PUFAs (47% in weight), with 38% as linoleic acid and 9% as α-linolenic acid (5). Because of their fatty acid composition, walnuts increase circulating concentrations of PUFAs, particularly linoleic acid and α-linolenic acid (13–16), which may favorably influence insulin resistance (17) and risk of type 2 diabetes (4). Walnuts also have high amounts of dietary fiber, antioxidants, and phytosterol (18, 19). Growing evidence from dietary intervention studies suggests beneficial effects of walnut consumption on lipid profile (20). In the meta-analysis by Banel et al. (20), walnut-rich dietary interventions significantly decreased total cholesterol by 0.27 mmol/L and LDL cholesterol by 0.24 mmol/L, without affecting HDL cholesterol and TGs. Despite concerns that high amounts of unsaturated fatty acids may promote oxidative stress, several intervention studies found that oxidative stress markers remained unchanged during walnut interventions despite increased intakes of PUFAs (21–23). In several feeding studies, markers of endothelial function, including vascular cell adhesion molecule 1, were significantly reduced by walnut diets compared with control diets. In other clinical trials, walnut diets improved markers of endothelial function (24–27), ameliorated central obesity and improved metabolic syndrome parameters (16), and increased circulating total adiponectin and apoA concentrations (28).

In the present study, we found that total nut consumption was associated with a lower risk of incident type 2 diabetes; however, the association was attenuated and became nonsignificant after controlling for BMI. Women with frequent nut consumption tended to be leaner than those who rarely consumed nuts at baseline (6), and previous studies in our cohorts revealed that frequent nut consumption was associated with less weight gain (7). Therefore, it is possible that body weight mediated the association between nut consumption and reduced risk of type 2 diabetes. Despite their high energy and high fat content, nut consumption does not appear to induce weight gain in many intervention studies (29) and may increase satiation (30). Cross-sectional and prospective cohort studies showed that nut consumption was related to a low risk of metabolic syndrome (31, 32). Several short-term intervention studies suggested beneficial effects of nut consumption on lipid profile (33, 34), inflammatory markers and endothelial function (35), oxidative stress (36, 37), insulin secretion (38), and glucose homeostasis (39), which may explain the inverse association between habitual nut consumption and risk of type 2 diabetes. It is worth noting that a higher consumption of different types of nuts, including almonds, walnuts, and peanuts, appears to have similar benefits on blood lipids (40). Although the inverse association observed for walnuts appeared to be stronger than for total nuts or other tree nuts, a formal comparison of the benefits of different types of tree nuts was not possible, because we did not specifically assess consumption of other tree nuts.

Recently, results from the PREDIMED randomized trial suggested that a Mediterranean diet supplemented with 30 g/d nuts (50% walnuts, 25% almonds, and 25% hazelnuts) significantly reduced risk of metabolic syndrome (41) and incidence of type 2 diabetes (42) compared with the low-fat control diet. Intervention studies found that 56 g/d of mixed nuts as a replacement for carbohydrate foods improved glycemic control in patients with type 2 diabetes (43). Several other clinical trials specifically used walnuts as the intervention food and found benefits on blood lipids (44) and endothelial function (27). This evidence supports a role of nut consumption in the prevention and management of diabetes.

The strengths of the current study include a large sample size, a prospective design, and repeated assessments of diet and lifestyle variables. Our study has several potential limitations. First, our study populations primarily consisted of white female nurses, which may limit the generalizability of the findings to other ethnic groups or males. Second, because diet was assessed by FFQs, measurement error of nut intake is inevitable, which may underestimate the true associations. Third, biochemical markers for type 2 diabetes (fasting glucose, insulin, lipids, and HbA1C, etc.) were not available in the full NHS cohorts, and thus could not be adjusted in the models. Furthermore, habitual nut consumption was associated with several healthy lifestyle practices and may be a marker for an overall healthy lifestyle. Although we carefully controlled for a number of diabetes risk factors, unmeasured and residual confounding is still possible to explain the association and we could not fully exclude the potential influence from the overall diet quality and healthy cuisine effects. Finally, although causation cannot be inferred from our analysis, the consistent evidence from experimental and epidemiological studies and clinical trials provides support for the hypothesis that regular consumption of nuts, particularly walnuts, as a component of a healthy diet profile is associated with lower incidence of type 2 diabetes.

In conclusion, the present analysis indicates that frequent intake of walnuts was associated with a lower risk of incident type 2 diabetes in women, the association persisted after adjustment for other lifestyle factors, and it was partially mediated by BMI. The inverse association between other tree nuts and total nuts with type 2 diabetes was largely explained by BMI. Further studies are needed to confirm our results, particularly the association between walnut consumption and risk of type 2 diabetes. The findings from our study and others support the benefits of the incorporation of nuts, including walnuts, as a component of a healthy diet for diabetes prevention.
